# Longitudinal association of type 1 interferon-induced chemokines with disease activity in systemic lupus erythematosus

**DOI:** 10.1038/s41598-018-20203-9

**Published:** 2018-02-19

**Authors:** K. L. Connelly, R. Kandane-Rathnayake, M. Huq, A. Hoi, M. Nikpour, E. F. Morand

**Affiliations:** 1 Centre for Inflammatory Diseases, School of Clinical Sciences at Monash Health, Faculty of Medicine, Nursing and Health Sciences, Monash University, 246 Clayton Road, Clayton, VIC 3168 Australia; 2 Department of Medicine and Rheumatology, The University of Melbourne at St. Vincent’s Hospital, Melbourne, Australia

## Abstract

Type I interferon (IFN) pathways are significant in SLE pathogenesis. Less is known about the utility of measuring markers of IFN activity in patients, or whether patient subsets with different profiles exist. We explored the longitudinal associations of IFN-induced chemokines with disease activity in a cohort of SLE patients. We calculated a validated composite score (IFN-CK) of three type I IFN-inducible chemokines (CCL2/CXCL10/CCL19) measured in 109 SLE patients (median 7 occasions over 3.2 years). Longitudinal associations of IFN-CK score with disease activity (SLEDAI-2K) and other variables were assessed using general estimating equation (GEE) methods. IFN-CK was detectable in all patients. SLEDAI-2K was significantly associated with IFN-CK, damage score and prednisolone dose. SLEDAI-2K remained significantly associated with IFN-CK over time after adjustment of covariates. Patients with high time-adjusted mean IFN-CK had lower complement and higher time-adjusted disease activity. Concordance between IFN-CK and SLEDAI-2K varied widely among patients, with some individuals having none, others weak, and a subset very high concordance. In summary in our cohort of SLE patients, serum IFN-CK varied over time with disease activity, but with wide variation in concordance. Differing relationships between IFN pathway activation and disease activity may be valuable in assigning patients to emerging IFN-pathway targeting treatments.

## Introduction

Systemic lupus erythematosus (SLE; lupus) is a chronic autoimmune disease characterised by immunologically-mediated inflammatory activity across multiple organ systems and the potential for irreversible end organ damage^1^. Striking clinical diversity is a hallmark of SLE, with variation in clinical manifestations, disease activity, and long term outcomes^1,2^. Similarly, a wide range of genetic, transcriptional, immunological and cellular events have been associated with SLE, but findings do not uniformly apply across patients. It is likely that this intrinsic biological diversity among patients has contributed to disappointing results in studies of promising targeted therapies, when applied to a pooled disease population^3,4^.

One of the best characterised biological pathways in SLE is the type I interferon (IFN) pathway, with finding of an IFN “gene signature”, a pattern of increased transcriptional activity of selected IFN-inducible genes, reported in a majority of SLE patients and replicated across multiple cohorts^5^. Given these associations, type I IFN is an attractive therapeutic target in SLE and multiple treatment strategies are under investigation^6^. A recent Phase II trial of anifrolumab, an anti-IFN receptor monoclonal antibody, found that responses were predicted by baseline IFN signature, suggesting a role for biological stratification in selecting patients for targeted therapies^7^. The association of changes in such signatures with disease activity is less clear^8^. In contrast, serum concentrations of certain chemokines induced by type I IFN that associate tightly with IFN-inducible gene expression^9,10^ have been reported to correlate with disease activity in previous studies^10,11^. A previous cross sectional study by the authors investigating the association of IFN-CK with disease parameters in SLE revealed findings including an association between IFN-CK score and disease activity^12^. Such previous studies have not included paired longitudinal serum biomarker levels with clinical data. It is not clear, therefore, whether variation in IFN-induced serum proteins vary with disease activity across time, or behave similarly in all patients.

The aim of the current study was to determine whether a composite index of type I IFN induced chemokines was associated with SLE disease activity over time, and particularly whether the strength of such relationships varied among patients, by using data from a large longitudinal set of paired clinical visits and serum samples. Our findings indicate a longitudinal association between type I IFN-induced chemokines and SLE disease activity, and identify the existence of divergence among patients according to the level of concordance between these measures. These findings suggest that measurement of IFN-induced chemokines over time may have utility in evaluating associations between disease activity and IFN pathway activation in SLE.

## Results

### Patient characteristics

Data from 944 visits in 109 patients were used in this analysis. Table 1 summarises the characteristics of the study population. In brief, 83% of the study group were female with a mean age at enrolment of 41.7 years (13.2). Half the subjects were of Asian ethnicity, with most others of European descendent. Median length of study follow-up was 3.2 years, and patients had a median of 7 clinic visits and matched serum samples during the study period. The time-adjusted mean SLEDAI-2K (AMS) of the study group was 4.4, with a TAM-PGA of 0.5. Patients used an average (TAM) prednisolone dose of 5.0 mg/day. Over 61% had organ damage (median SDI = 1) and 75% experienced flares during the study period. Assessed using SLEDAI-2K domains, serological activity was the most common manifestation of active disease (88%) followed by cutaneous (64%) and renal (42%) activity.

**Table 1:** Characteristics of study population.

Demographics	No. of patients (N = 109)
	**mean (SD)**
Age at enrolment (years)	41.7 (13.2)
	median [IQR] (range)*
Disease duration (years)	9.6 [4.6, 16.6] (1.6, 33.6)
Total follow-up period (years)	3.2 [2.4, 4.0] (0.2, 4.6)
Number of visits	7 [5, 11] (3, 27)
Number of ACR criteria fulfilled^1^	5 [4, 6] (4, 9)
TAM^2^ SLEDAI-2K^3^	4.4 [2.4, 6.7] (0, 15.1)
TAM PGA^4^	0.5 [0.3, 0.9] (0, 2.1)
TAM Prednisolone (mg/day)	5.0 [1.2, 8.6] (0, 33.6)
Cumulative PNL (mg)	3378 [595, 6318] (0, 50820)
SLICC SDI score (organ damage index)^5^	1 [0, 2] (0, 9)
TAM C3 (g/L) (mean (SD)) TAM C4 (g/L) (mean(SD)) TAM IFN-CK score^6^	0.9 (0.3) 0.2 (0.1) 0.3 [0.2, 0.5] (0.1, 2.9)
	**n (%)**
Females	90 (83%)
Ethnicity	
non-Asians	58 (53%)
Asians	51 (47%)
Anti-dsDNA positivity ever	82 (75%)
Organ damage (SLICC-SDI > 0)	67 (62%)
Flares ever (mild/mod./severe)	81 (74%)
Organ specific manifestations	
CNS	10 (9%)
Vasculitis	5 (5%)
Musculoskeletal	35 (32%)
Renal	46 (42%)
Cutaneous	70 (64%)
Serositis	8 (7%)
Serological	96 (88%)
Fever	1 (1%)
Haematological	21 (19%)

^*^Except as noted.

^1^Number of ACR criteria fulfilled at enrolment to the Monash SLE clinic; ^2^TAM = time adjusted mean; ^3^SLEDAI-2K score ranges from 0 to 105 and higher scores mean high disease activity; ^4^PGA score ranges from 0 to 3; ^5^SLICC SDI score ranges from 0 to 44 and high score mean more organ damage; ^6^IFN-CK score ranges from 0 to 3.

### Longitudinal associations of disease activity

Univariable GEE analyses showed several factors to be statistically significantly associated with SLEDAI-2K at each visit, as shown in Table 2. Increasing age was associated with a small but significant reduction in SLEDAI-2K. Both PGA and SDI score were positively associated with an increase in SLEDAI-2K, as was prednisolone dose; an increase of dose by 10 mg/d was associated with an increase of disease activity by 1.1 SLEDAI-2K units. We also observed a positive association between IFN-CK score and SLEDAI-2k. An increase of one unit in IFN-CK score was significantly associated with an increase in SLEDAI-2K of 0.7 (RC = 0.73, (95% CI: 0.12, 1.43) p = 0.02). Gender and ethnicity were not statistically significantly associated with disease activity. After adjustment using multivariable GEE analysis, prednisolone dose, PGA, SDI, and age remained statistically significantly associated with SLEDAI-2K (Table 3). After adjustment, IFN-CK score also remained significantly associated with SLEDAI-2K, wherein one unit increase in IFN-CK was associated with a SLEDAI-2K increase of 0.5 ((95% CI: 0.04, 0.98), p-value = 0.03). This method confirms the longitudinal association of IFN-CK with disease activity in SLE.

**Table 2:** Univariable associations of SLEDAI-2K determined using generalised estimating equation (GEE) method with covariates at each visit (n = 944 visits from 109 patients).

	RC	(95% CI)	p-value
Age at enrolment (years)	−0.07	(−0.11,−0.02)	p < 0.01
Disease duration (years)	0.04	(−0.04,0.12)	0.3
PGA^1^	2.59	(2.11,3.06)	p < 0.01
SLICC SDI score^2^	0.48	(0.19,0.76)	p < 0.01
Prednisolone (mg/d)	0.11	(0.08,0.14)	p < 0.01
IFN-CK score^3^	0.73	(0.12,1.34)	0.02
Female gender	0.01	(−1.55,1.58)	0.9
Asian ethnicity	0.78	(−0.34,1.91)	0.17

^1^PGA ranges from 0 to 3; ^2^SLICC SDI score ranges from 0 to 44; ^3^IFN-CK score ranges from 0 to 3.

RC: regression coefficient (exponentiated beta coefficient).

CI: confidence interval.

**Table 3:** Multivariable associations of SLEDAI-2K determined using generalised estimating equation (GEE) method (n = 939 visits from 109 patients).

	RC	(95% CI)	p-value
IFN-CK score^1^	0.55	(0.08, 1.01)	0.02
Age at enrolment (years)	−0.05	(−0.08, −0.01)	0.01
Prednisolone (mg/d)	0.07	(0.04, 0.10)	p < 0.01
PGA^2^	2.31	(1.84, 2.78)	p < 0.01
SLICC SDI score^3^	0.31	(0.09, 0.52)	0.01

^1^IFN-CK scores ranged from 0 to 3; ^2^PGA ranged from 0 to 3; ^3^SLICC SDI ranged from 0 to 44.

RC: regression coefficient.

CI: confidence interval.

### Comparison of low and high IFN-CK groups

We next compared clinical characteristics between patients with high and low time-adjusted mean IFN-CK scores. Low IFN-CK was deemed ≤0.3 and high IFN-CK > 0.3 based on the median value of all time-adjusted mean IFN-CK scores. Serum complement levels were significantly lower in the high IFN-CK group (Table 4). As expected given the results above, there was also numerically greater disease activity over time in the high IFN-CK group, with a median AMS in the high IFN-CK group of 5.2 compared with 3.6 in the low IFN-CK group (p = 0.06). Frequency of dsDNA positivity was also numerically higher in the high IFN-CK group (83% vs. 68%, p = 0.07).

**Table 4:** Comparison of patient characteristics between low and high TAM-IFN-CK groups.

	TAM_IFNCK < 0.3	TAM_IFNCK ≥ 0.3	p-value
	(n = 56)	(n = 53)	
	**mean (SD)**	**mean (SD)**	
Age at enrolment (years)	43.2 (14.0)	40.1 (12.3)	0.2
	median [IQR]*	median [IQR]*	
Disease duration (years)	8.1 [4.6, 16.1]	9.6 [5.6, 16.6]	0.6
Total follow-up period (years)	3.1 [2.3, 4.0]	3.3 [2.6, 4.2]	0.6
Number of visits	7 [5, 11]	8 [5, 11]	0.5
Number of ACR criteria fulfilled^1^	5 [4, 6]	5 [4, 7]	0.2
TAM^2^ SLEDAI-2K^3^	3.6 [2.2, 5.4]	5.2 [2.8, 6.9]	0.06
TAM PGA^4^	0.5 [0.3, 0.9]	0.6 [0.3, 0.9]	0.3
TAM Prednisolone (mg/day)	5.0 [0.0, 9.1]	5.2 [2.8, 8.4]	0.4
Cumulative PNL (mg)	2450 [0, 6048]	3815 [1330, 6388]	0.3
TAM C3 (g/L) (mean(SD)) TAM C4 (g/L) (Mean(SD)) SLICC SDI score (organ damage index)^5^	0.9 [0.3] 0.2 [0.1] 1 [0, 2]	0.8 [0.3] 0.1 [0.1] 1 [0, 2]	0.02 0.04 0.5
	**n (%)**	**n (%)**	
Female	47 (84%)	43 (81%)	0.7
Asian ethnicity	24 (43%)	27 (51%)	0.4
Anti-dsDNA positivity ever	38 (68%)	44 (83%)	0.07
Organ damage (SLICC-SDI > 0)	32 (57%)	35 (66%)	0.3
Flares ever	40 (71%)	41 (77%)	0.5
Organ specific manifestations			
CNS	5 (9%)	5 (9%)	0.9
Vasculitis	2 (4%)	3 (6%)	0.6
Musculoskeletal	15 (27%)	20 (38%)	0.2
Renal	20 (36%)	26 (49%)	0.2
Cutaneous	36 (64%)	34 (64%)	0.9
Serositis	3 (5%)	5 (9%)	0.4
Serological	47 (84%)	49 (92%)	0.2
Fever	1 (2%)	0 (0%)	0.3
Haematological	8 (14%)	13 (25%)	0.2

*Except as noted

^1^At enrolment to the Monash SLE clinic; ^2^TAM = time adjusted mean; ^3^SLEDAI-2K score ranges from 0 to 105 and higher scores means high disease activity; ^4^PGA score ranges from 0 to 3; ^5^SLICC SDI scores range from 0 to 44 and high scores mean more organ damage.

*P-values were derived using t-test, Wilcoxon rank-sum test and Pearson’s chi-squared tests to compare means, medians and percentages respectively.

### Concordance between IFN-CK and SLEDAI in individual patients

**Figure 1:**
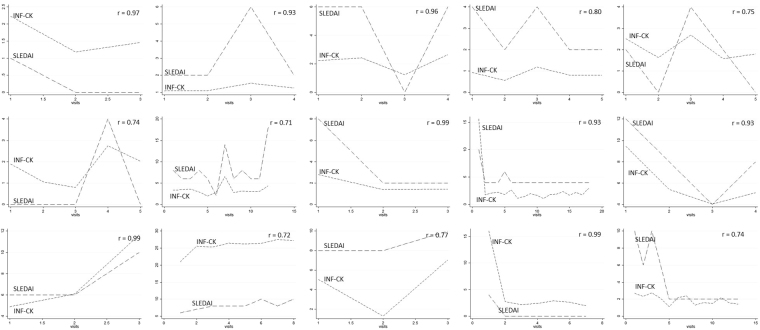
IFN-CK SLEDAI-2K concordance in patients with r ≥ 0.7. Concordance between type I interferon inducible chemokine score (IFN-CK) and disease activity (SLEDAI-2k) was assessed in individual patients. A strong concordance between these variables over time (r ≥ 0.7) was seen in a subset of 15 patients.

**Figure 2:**
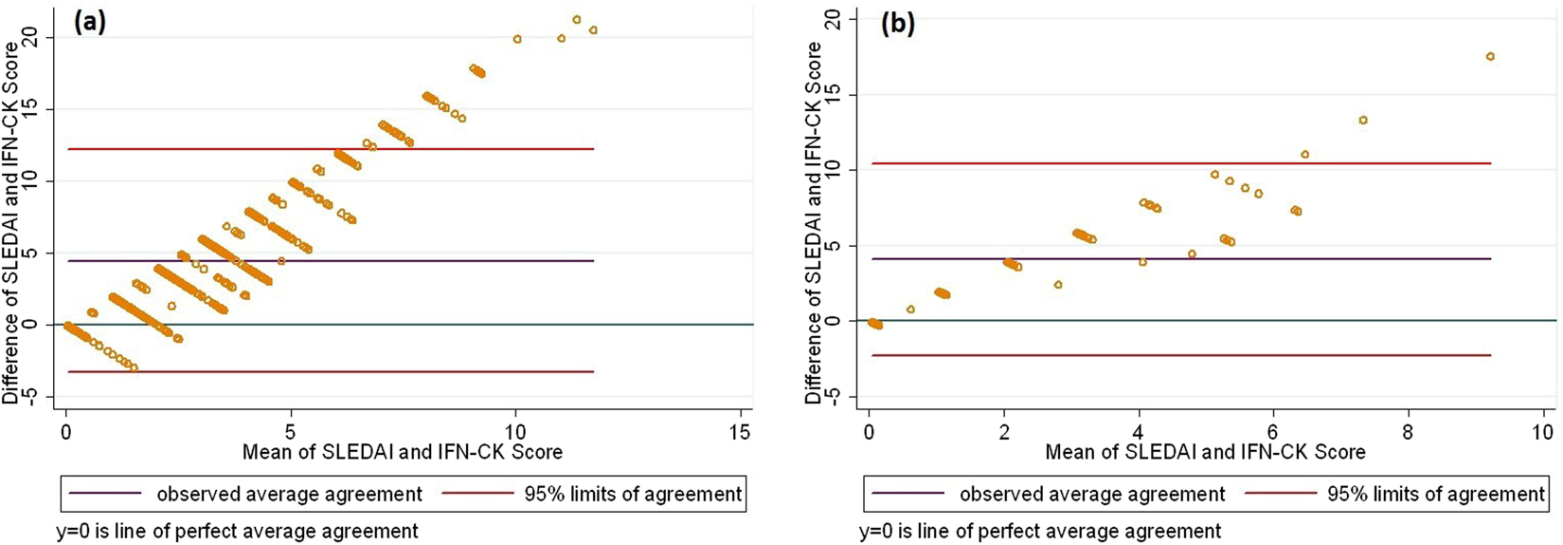
Bland-Altman’s limits-of-agreement for (**a**) overall study population; (**b**) patient group with correlation coefficient r ≥ 0.7 for IFN-CK and SLEDAI-2K. Bland-Altman graphs were generated to examine the extent of agreement between type I interferon inducible chemokine score (IFN-CK) and disease activity (SLEDAI-2k) in the overall study population (graph 2a) and in the patient group with correlation coefficient r ≥ 0.7 for IFN-CK and SLEDAI-2k (graph 2b). Greater concordance was seen for lower values of SLEDAI-2k and IFN score.

When plotting individual patient time series, it was noticeable that some patients had concordance over time between IFN-CK and SLEDAI-2K scores, while others did not. In order to categorise patients according to IFN-CK:SLEDAI-2K concordance, a correlation coefficient for each patient was calculated. Of the 109 patients, 7 patients had identical SLEDAI-2K scores at all visits, therefore correlation coefficients were not calculated. Of the remaining 102 patients, 39 (38%) had a correlation coefficient (r) less than or equal to zero (r ≤ 0). Of the 63 patients with r > 0, 15 patients had r ≥ 0.7, demonstrating strong concordance between IFN-CK and SLEDAI-2K (Fig. 1). Interestingly, Bland-Altman graphs, used to examine the extent of agreement between two variables by plotting the differences between the pairs of measurements against the mean of each pair, indicated greater concordance for lower values of SLEDAI-2K and IFN-CK score, even among patients where these was a strong correlation over time between these variables (r ≥ 0.7) (Fig. 2).

We further investigated whether patients in this sub-group differed from the groups with r ≤ 0 and those with r > 0 but < 0.7). No differences were observed in standard indicators of disease activity and severity such as AMS, PGA, or number of ACR criteria. However, patients in the high correlation group (r ≥ 0.7) had significantly fewer visits and lower time adjusted mean and cumulative prednisolone doses (Table 5).

**Table 5:** Patient characteristics by IFN-CK SLEDAI-2K concordance categories.

	IFN-CK SLEDAI-2K concordance categories			p-value*
	r < = 0 (n = 39)	0 < r < 0.7 (n = 48)	r > = 0.7 (n = 15)	
	**mean (SD)**	**mean (SD)**	**mean (SD)**	
Age at enrolment (years)	40.7 (13.6)	42.4 (11.4)	38.9 (16.7)	0.6
	median [IQR]*	median [IQR]*	median [IQR]*	
Disease duration (years)	7.6 (3.6, 15.6)	11.6 (6.1, 16.1)	7.6 (4.6, 19.6)	0.2
Number of visits	8.0 (5.0, 11.0)	8.5 (6.0, 11.5)	5.0 (3.0, 8.0)	0.02
Number of ACR criteria fulfilled^1^	5.0 (4.0, 6.0)	5.0 (4.0, 6.0)	5.0 (4.0, 6.0)	0.9
TAM^2^ SLEDAI-2K^3^	5.0 (2.4, 6.3)	3.7 (2.4, 7.2)	3.9 (2.3, 6.8)	0.8
TAM PGA^4^	0.5 (0.3, 1.0)	0.6 (0.3, 0.9)	0.5 (0.3, 1.0)	0.9
TAM Prednisolone (mg/day)	5.3 (1.2, 9.2)	5.6 (3.6, 10.5)	2.3 (0.0, 5.5)	0.04
Cumulative PNL (mg)	3780 (595, 5845)	5110 (1863.8, 10745)	1421 (0, 3990)	0.03
SLICC SDI score (organ damage)^5^	1.0 (0.0, 2.0)	1.0 (0.0, 2.5)	1.0 (0.0, 2.0)	0.6
TAM C3 (g/L) (mean(SD)) TAM C4 (g/L) (mean(SD)) TAM IFN-CK score^6^	0.8 (0.3) 0.2 (0.1) 0.2 (0.2, 0.8)	0.9 (0.3) 0.2 (0.1) 0.4 (0.2, 0.7)	1.0 (0.3) 0.2 (0.1) 0.2 (0.1, 0.4)	0.18 0.6 0.3
	**n (%)**	**n (%)**	**n (%)**	
Females	32 (82%)	43 (90%)	11 (73%)	0.3
Asian ethnicity	19 (49%)	25 (52%)	4 (27%)	0.2
Anti-dsDNA positivity ever	31 (79%)	37 (77%)	10 (67%)	0.6
Organ damage (SLICC-SDI > 0)	24 (62%)	30 (63%)	8 (53%)	0.8
Flares ever (mild/mod./severe)	33 (85%)	35 (73%)	13 (87%)	0.3

*except as noted.

^1^Number of ACR criteria fulfilled at enrolment to the Monash SLE clinic; ^2^TAM = time adjusted mean; ^3^SLEDAI-2K score ranges from 0 to 105 and higher scores mean high disease activity; ^4^PGA score ranges from 0 to 3; ^5^SLICC SDI score ranges from 0 to 44 and high scores mean more organ damage; ^6^IFN-CK scores range from 0 to 3.

*P-values were derived using ANOVA, Kruskal Wallis test and Pearson’s chi-squared tests to compare means, medians and percentages respectively.

## Discussion

Serum concentrations of IFN-induced chemokines are a surrogate marker of activation of the type I IFN system, which has been strongly implicated in the pathogenesis of SLE. IFN-induced gene transcriptional signatures measured in peripheral blood have been most often used to interrogate the IFN system in human SLE, but the most thorough longitudinal studies to date have not demonstrated strong associations of IFN gene signatures with disease activity over time^8^. Using a validated composite score derived from serum concentrations of IFN-induced chemokines, in a prospectively-followed multi-ethnic lupus cohort, we have demonstrated a significant longitudinal association between this marker of type I IFN activity and SLE disease activity. Moreover, we demonstrate that the strength of the relationship between disease activity and IFN-CK varies markedly between patients, with some patients showing high, and others no, concordance between these variables.

While several studies have suggested an association between the type I IFN system and SLE disease activity, very few have had the longitudinal design necessary to investigate fluctuations with disease activity in patients over time. In a large longitudinal study of paediatric SLE, no strong associations of IFN transcriptional signatures with disease activity over time were found^8^. We have here demonstrated, using the GEE method, that an increase in type I IFN activity as measured by IFN-CK score is associated with an increase in SLEDAI, and this association remains significant on multivariable analysis adjusting for other variables associated with disease activity. This adds support to prior observations unadjusted for such confounders which suggested that patients with high IFN-induced chemokines at baseline were more likely to flare over the subsequent year, and that IFN-CK score may rise with disease flares^11^. The current study advances on our previous work associating IFN-CK with increased disease activity, by analysing paired longitudinal biomarker and clinical data, better reflecting the association between fluctuations among patients in IFN-CK score and SLEDAI, while accounting for correlations within patients.

Our categorical analysis also suggested patients in the high IFN-CK group had higher markers of disease activity over time, including higher AMS, reduced complement levels and higher anti-dsDNA. Serological markers of disease activity such as complement (C3 and C4) have been found to inversely correlate with type I IFN activity in SLE in previous studies of IFN-induced gene transcripts^13,14^. The biological basis of this association may relate to the induction of the type I IFN system by immune complexes, neutrophil extracellular traps or other endogenous stimuli in SLE^15^. Type I IFN transcriptional signatures have also been strongly linked to renal and to a lesser extent CNS and haematological manifestations in SLE^16^. In our study IFN-CK scores had the strongest association with overall SLEDAI-2k rather than individual organ manifestations, although cutaneous and haematological manifestations were the strongest contributors to this association (data not shown). This discrepancy in findings between studies may be due to the IFN gene signature and IFN induced serum chemokines representing two slightly different measures of the type I IFN system^17^. Gene transcription peripheral blood signatures appear to be a more static measure, suitable for predicting disease phenotype even though not associated with disease activity over time^8,18–20^. Consistent with this, standard doses of corticosteroids which reduce disease activity do not suppress the IFN signature^21^.

In addition to confirming a longitudinal association between IFN-CK score and SLEDAI in an overall cohort, our study also identified the important finding that patients vary widely in their concordance between these variables, with the rise and fall of these variables mirroring one another closely in some patients but not at all in others. Interestingly, concordance appeared to be greater in patients with lower IFN-CK scores and less active disease. One possible explanation is the effect of glucocorticoids on the expression of CCL2, CCL19, and CXCL10 in response to type I IFN. The induction of these chemokines by Type I IFN is mediated by promoters that are highly sensitive to glucocorticoids, such as nuclear factor kappa B^22,23^. In a previous study investigating the serum IFN-CK score used here, patients with inactive disease had significantly lower IFN-CK scores when on high doses of prednisolone (≥10 mg daily) compared with low doses or no prednisolone^24^. In our study, doses of prednisolone were significantly lower in the high concordance group. Increased doses of prednisolone could therefore have impacted on the ability to discern correlations with IFN-CK in higher disease activity groups.

Importantly, markers that relate IFN system activity to disease activity may be of utility in assigning patients to treatment with emerging anti-IFN-pathway drugs. In the Phase II trial of anti-IFN receptor monoclonal antibody anifrolumab, IFN gene signature was used to biologically stratify patients, with a high gene signature predicting response to therapy^25^. This raises interest in the role of a more downstream marker of type I IFN activity, such as IFN-CK, in such trials. Given that IFN-CK (in contrast to IFN gene signature) appears to correlate not only with IFN activity but also fluctuations in disease activity, such a marker conceivably has utility in stratifying patients and monitoring response to treatment.

A unique feature of our cohort is its ethnic composition, with close to half of all patients being of Asian background. While type I IFN activity has been confirmed in Asian SLE patients using IFN induced gene transcripts^9,26^, previous studies of IFN-induced chemokines have been limited mainly to patients of Caucasian, African American and Hispanic ethnicity. We recently reported a cross-sectional study of serum IFN-CK in SLE in which the association of Asian ethnicity with higher disease activity was independent of IFN-CK^12^. Given this it is interesting to note that Asian patients represented only 27% of those with high concordance between IFN-CK and SLEDAI, despite comprising close to 50% of the overall cohort. This lends further support to the hypothesis that type I IFN may be a less predominant biological driver of disease activity, or that IFN-CK less well reflects IFN pathway activation, in SLE in Asians compared to other ethnicities. This is also the longest-duration study to date investigating associations between type I IFN and disease activity in SLE, and was performed using a serum archive tightly linked to prospectively acquired disease activity data. Limitations of the current study include being limited to 109 patients; a larger cohort may have allowed demonstration of additional significant associations of IFN-induced chemokines.

In conclusion, we have confirmed a longitudinal association between serum concentration of type I IFN-induced chemokines and disease activity in SLE. Importantly, Type I IFN induced chemokines were detectable in 100% of patients, in contrast to many other cytokines that are only detectable in a subset of cases^27,28^. Our data also suggest the existence of subgroups of patients with widely variable concordance between IFN-CK score and SLEDAI, indicating that the relationship between IFN-CK and disease activity is present only in some patients. These data lend further support to the potential of type IFN-induced serum pro-inflammatory proteins, as opposed to IFN-induced gene signatures, to identify the subgroup of patients with both active disease and activation of the Type I IFN system. These findings may be relevant to the stratification of patients undergoing therapy with treatments targeting the Type I IFN system.

## Methods

### Study design and participants

Data were prospectively acquired between June 2007 and January 2012 from patients who attended the SLE Clinic at Monash Medical Centre, a tertiary referral public hospital in Melbourne, Australia, who fulfilled the American College of Rheumatology (ACR) criteria for the classification of SLE^29^, were over 18 years of age, had complete data available and provided written informed consent^27,30^. Patients were included in the current study if they had complete clinical data and a matched serum sample available for at least three separate clinic visits. Ethics approval for this study was obtained from, and the study carried out in accordance with, the Monash Health Human Research Ethics Committee.

### Patient information

Patients were seen at 3–6 monthly intervals, or more frequently according to clinical need. At each clinic visit disease activity was documented using the 2000 modification of the SLE disease activity index (SLEDAI-2K)^31^. A measure of disease activity over time was generated using the adjusted mean SLEDAI-2K (AMS)^31^. Disease-related damage was assessed at baseline and annually using the Systemic Lupus International Collaborating Clinics (SLICC) Damage Index (SDI)^32^. Birth date, gender, year of disease onset and ethnicity were recorded at baseline. Autoantibody positivity was documented at baseline and included ANA titre, anti-double stranded DNA (anti-dsDNA) positivity and antibodies to a range of extractable nuclear antigens (ENA) including ribonucleoprotein (RNP), Sm, Ro, and La.

### Measurement of serum concentrations of IFN induced chemokines (IFN-CK)

Patient serum samples were obtained and stored at −80^0^C until use as described^12,27,28^. Activation of type I IFN pathways was assessed by measurement of three type I IFN inducible chemokines (CCL2, CXCL10 and CCL19) as described by Bauer^11^. Concentrations of serum CCL2, CXCL10 and CCL19 were determined in each sample using sandwich ELISA, as previously described^12,27^. Briefly, 96-well plates (Immunoplates, Nunc, Roakilde, Denmark) were coated with primary antibody (anti-human CCL2, CXCL10 or CCL19; R&D Systems, Minneapolis, MN, USA) and incubated overnight before being blocked by 1% bovine serum albumin. After washing, recombinant human protein standards and serum samples were added in duplicate and incubated overnight. Binding was detected using a biotinylated goat anti-human antibody (R&D Systems) and streptavidin conjugated to horseradish peroxidase (Silenus, Melbourne, Australia). Colour was developed with 3,3′5,5′-tetramethylbensidine (Sigma, Sydney, Australia) and read at 450 nm. In order to integrate the results obtained for the three type I IFN induced chemokines, a composite IFN-CK score was derived for each sample, in the manner validated by Bauer *et al*.^11^: concentrations above the 95th centile for each chemokine were assigned a value of one, with the remaining concentrations scaled to this percentile. Scaled values for each chemokine were then added to produce a final IFN-CK score ranging from 0 to 3.

### Statistical Analysis

Statistical analyses were performed using Stata version 14 (StataCorp, College Station, Texas, USA). Continuous variables were described either as mean (standard deviation (SD)) or median (interquartile range [IQR], range) according to data distribution; categorical variables were described as frequency (%). Time adjusted means were calculated for several continuous variables to account for varying time intervals between visits. High time adjusted mean IFN-CK was defined as a score above the median value of 0.3. Several factors including demographics and disease characteristics were compared between low (<0.3) and high time adjusted means IFN-CK (≥0.3) and p-values were derived using t-tests, Wilcoxon rank sum tests and Pearson’s chi-squared tests to compare means, medians and percentages respectively.

The generalised estimating equation (GEE) method was used to examine longitudinal associations of SLEDAI-2K with several variables measured repeatedly (e.g. complement, PGA, IFN-CK). This is in contrast to previous cross-sectional studies where associations between IFN-CK and disease activity were investigated using linear and logistic regression models^12^. The GEE approach specifies how the outcome of a subject changes with covariates from one measurement to the next, while allowing for the correlation between repeated measurements on the same subject over time. Since SLEDAI-2K is a continuous variable, we specified Gaussian distribution for the family along with an identity link, and exchangeable correlation matrix in the model. Robust standard errors were derived adjusting for patient clustering. The QIC (quasilikelihood under the independence model criterion) method was used to assess the best working correlation structures and best subsets of covariates for GEE analyses. Univariable GEE models were performed for each independent variable and a p-value threshold of 0.1 was applied for variable selection for the multivariable model. Potential collinearity between independent variables was also assessed before including these in the multivariable model. Results were reported as regression coefficients (RC, exponentiated beta coefficients) with corresponding 95% confidence intervals (95% CI). A p-value < 0.05 was considered statistically significant.

In addition, we determined the concordance between IFN-CK and SLEDAI-2K in individual study participants using Pearson’s correlation coefficients (r). Based on the r values, patients were grouped in to three categories: r < 0 (no correlation); 0 < r < 0.7 (intermediate correlation), and r >  = 0.7 (strong correlation). We used Bland-Altman plots to determine the degree of concordance between SLEDAI-2K and IFN-CK throughout the range of possible values for these variables, in patients where the correlation between these variables was strong (r > 0.7). Patient characteristics were compared among these three categories: means were compared using ANOVA, medians were compared using the Kruskal-Wallis test and proportions were compared using Pearson’s chi-Squared test.
